# Metagenomic and metatranscriptomic analysis of saliva reveals disease-associated microbiota in patients with periodontitis and dental caries

**DOI:** 10.1038/s41522-017-0031-4

**Published:** 2017-10-02

**Authors:** Daniel Belstrøm, Florentin Constancias, Yang Liu, Liang Yang, Daniela I. Drautz-Moses, Stephan C. Schuster, Gurjeet Singh Kohli, Tim Holm Jakobsen, Palle Holmstrup, Michael Givskov

**Affiliations:** 1 Section for Periodontology and Oral Microbiology, Department of Odontology, Faculty of Health and Medical Sciences, University of Copenhagen, Copenhagen, Denmark; 2 Singapore Centre for Environmental Life Sciences Engineering, Nanyang Technological University Singapore, Singapore, Singapore; 3 Costerton Biofilm Center, Department of Immunology and Microbiology, Faculty of Health and Medical Sciences, University of Copenhagen, Copenhagen, Denmark

## Abstract

The taxonomic composition of the salivary microbiota has been reported to differentiate between oral health and disease. However, information on bacterial activity and gene expression of the salivary microbiota is limited. The purpose of this study was to perform metagenomic and metatranscriptomic characterization of the salivary microbiota and test the hypothesis that salivary microbial presence and activity could be an indicator of the oral health status. Stimulated saliva samples were collected from 30 individuals (periodontitis: *n* = 10, dental caries: *n* = 10, oral health: *n* = 10). Salivary microbiota was characterized using metagenomics and metatranscriptomics in order to compare community composition and the gene expression between the three groups. *Streptococcus* was the predominant bacterial genus constituting approx. 25 and 50% of all DNA and RNA reads, respectively. A significant disease-associated higher relative abundance of traditional periodontal pathogens such as *Porphyromonas gingivalis* and *Filifactor alocis* and salivary microbial activity of *F*. *alocis* was associated with periodontitis. Significantly higher relative abundance of caries-associated bacteria such as *Streptococcus mutans* and *Lactobacillus fermentum* was identified in saliva from patients with dental caries. Multiple genes involved in carbohydrate metabolism were significantly more expressed in healthy controls compared to periodontitis patients. Using metagenomics and metatranscriptomics we show that relative abundance of specific oral bacterial species and bacterial gene expression in saliva associates with periodontitis and dental caries. Further longitudinal studies are warranted to evaluate if screening of salivary microbial activity of specific oral bacterial species and metabolic gene expression can identify periodontitis and dental caries at preclinical stages.

## Oral health: Signals from gene activity in saliva

Genetic analysis of saliva reveals the activity of bacteria linked to gum disease and tooth decay and may prove useful in early diagnosis. Daniel Belstrøm and colleagues at the University of Copenhagen, Denmark, with co-workers at Nanyang Technological University in Singapore, analyzed saliva from 10 patients with periodontitis gum disease, 10 with dental caries and 10 with good oral health. DNA analysis revealed which bacteria were present, while examining RNA revealed which bacterial genes were most active. The procedure identified greater abundance and activity of bacteria linked to each specific oral condition in the oral disease groups, and also found distinctive bacterial activity in those people with good oral health. Further studies should investigate the possibility of testing bacterial gene activity in saliva to identify oral diseases before they become clinically evident.

## Introduction

Periodontitis and dental caries are the two most prevalent oral diseases and the primary causes of tooth loss in the western world.^1,2^ At present, periodontitis and dental caries are mostly diagnosed at late stages of disease, often leading to costly and invasive dental treatment. Therefore, new diagnostic approaches capable of identifying periodontitis and dental caries at preclinical stages, favoring preventive treatment strategies, are urgently needed.

The oral cavity harbors a diverse microbiota comprising more than 700 unique bacterial species.^3^ The microbiota plays a pivotal role in maintenance of oral homeostasis, as various oral habitats are colonized by characteristic bacterial community profiles organized in local biofilms.^4^ However, ecological changes, for example in relation to increased sugar intake, insufficiently performed oral hygiene or fluctuations in the immune response can induce structural^5–7^ and functional alterations^8–10^ of local oral biofilms. Such alterations may in turn change the relation between the host and the resident microbiota from symbiosis to dysbiosis, thereby fueling initiation and progression of periodontitis and dental caries.

Saliva is the biological fluid of the oral cavity which is critical for maintenance of oral and general health.^11^ Therefore, saliva has been intensively investigated for candidate biomarkers associated with oral health and disease.^12,13^ Saliva is sterile when entering the oral cavity,^14^ but when sampled, saliva contains a diverse microbiota.^15^ In healthy oral conditions, the composition of the salivary microbiota is different from that of supragingival and subgingival biofilms.^16^ On the other hand, the presence of specific bacterial species in saliva such as *Porphyromonas gingivalis* and *Streptococcus mutans* has been reported in individuals with periodontitis and dental caries, respectively.^17,18^ Essentially, these findings suggest that bacteria from local periodontitis and caries lesions may be spilled-over and dispersed into saliva. However, it remains unclear if dispersed bacteria remain metabolically active as they are translocated from the local ecological niche of the biofilms to saliva, which possesses different ecological properties.

So far, only a few studies have reported higher expression of specific bacterial genes to associate with dental caries.^19,20^ In addition, the potential of using comprehensive metatranscriptomic microbial analysis of saliva to identify possible biomarkers has not yet been evaluated.

The aims of the present study were to employ metagenomic and metatranscriptomic analyses to (i) characterize the diversity and community composition of the salivary microbiota, (ii) to utilize these data to determine the microbial activity (DNA/RNA ratio) of each taxon identified and, (iii) to identify differentially expressed microbial genes. The main hypothesis was that the taxonomic composition, as well as microbial activity could be an indicator of the oral health status, and possible differences might potentially be used as proxy biomarkers of oral health and disease.

## Results

### General information

From a total of 30 saliva samples 768.62 M (mean 25.62 M; range 14.61 M–32.64 M) DNA sequences and 981.48 M (mean 32.72 M; range 26.40 M–38.44 M) RNA sequences were generated out of which 274.17 M (DNA; mean 9.14 M: range 2.58 M–18.52 M) and 148.49 (RNA; mean 4.50 M; range 2.93 M–9.11 M) passed quality control (Supporting material Table S1).

**Fig. 1:**
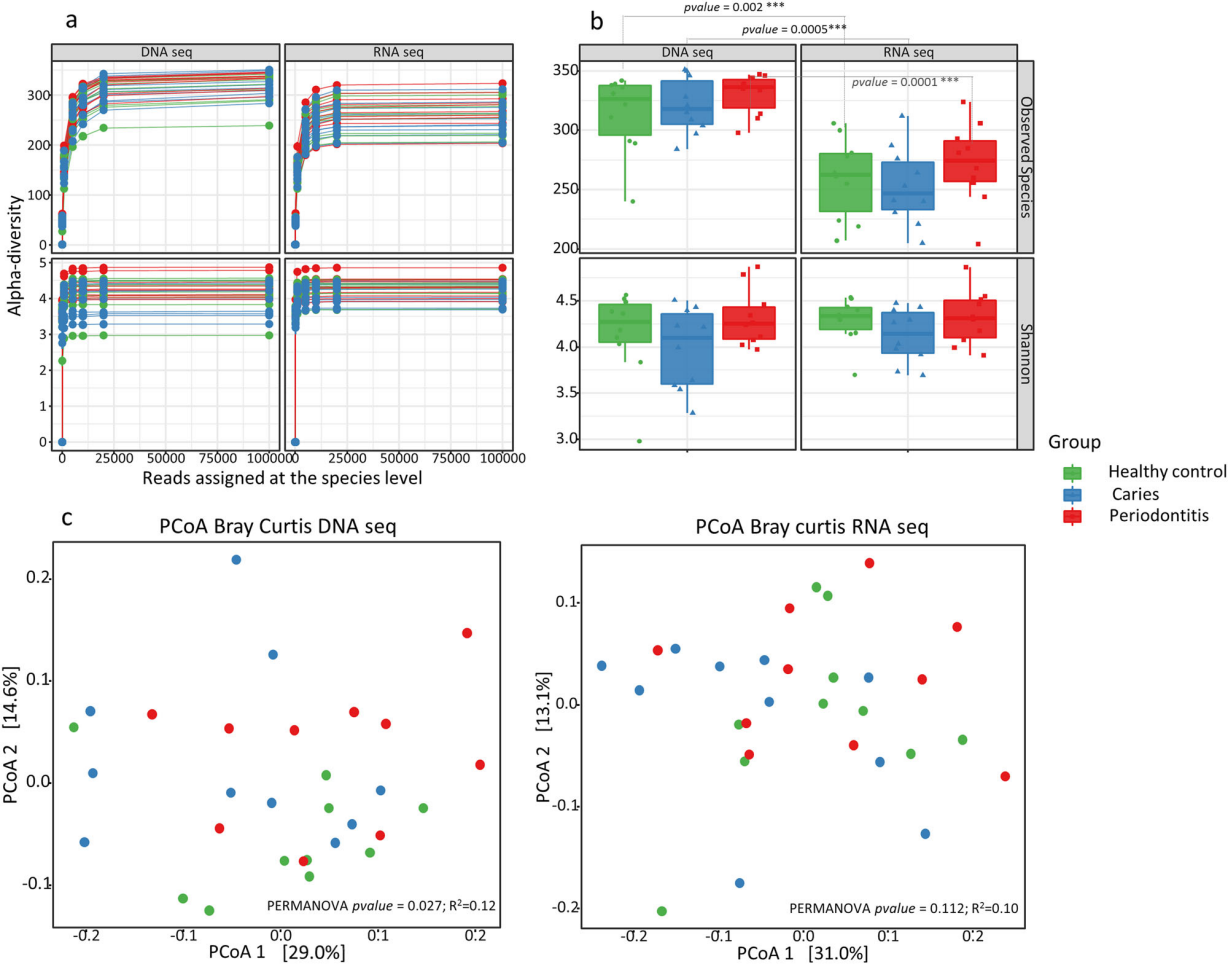
Microbial diversity. **a**: Rarefaction curves expressed as number of reads assigned at species level (*x*-axis) and α-diversity (observed Species and Shannon index, *y*-axis) of salivary microbiota characterized using metagenomics and metatranscriptomics. **b**: Distribution of α-diversity among groups presented as boxplot (median, lower/upper quartile and standard deviation). **c**: β-diversity determined by Bray-Curtis dissimilarity and plotted using PCoA. Sample denotation: green: healthy controls, blue: dental caries, red: periodontitis

At species level, a significantly higher α-diversity (observed species) was recorded by metagenomics compared to metatranscriptomic analysis (Figs. 1a–b) (*p* < 0.05). However, no difference in α-diversity was observed between groups using either metagenomics or metatranscriptomic approaches. Metagenomic analysis yielded a significantly different β-diversity between groups (*p* = 0.027, *R* ^2^ = 0.12), whereas an insignificantly different β-diversity was recorded when employing metatranscriptomic analysis (*p* = 0.11, *R* ^2^ = 0.10) (Fig. 1c).

A total of 810 to 894 microbial KEGG Orthologs (KO) was identified in salivary microbial metagenomes and metatranscriptomes. These KO were mainly associated to Ribosome, Carbon metabolism, ABC transporter, and Purine metabolism pathways (Supporting material Fig S1A). Within the metabolism category salivary microbiomes were dominated by formate C-acetyltransferase, enolase, 5-methyltetrahydropteroyltriglutamate--homocysteine methyltransferase, and pyruvate oxidase (Supporting material Fig S2A). In all cases, a significantly lower number of KEGG KO and Pathways were identified in the salivary metagenomes as compared to the microbial metatranscriptome (*p* < 0.05, Supporting material Figs S1B, S2B). In order to characterize the overall community differences in terms of KEGG pathways and KO, Bray–Curtis overall dissimilarities were constructed and no significant discrimination of healthy and disease groups were observed (PERMANOVA *p* > 0.05 in all case, Supporting material Figs S1 C-D; Fig S2 C-D).

### Identification of microbial taxa from salivary metagenomes and metatranscriptome

A total number of 12 bacterial phyla, 123 bacterial genera, and 351 bacterial species were identified from the salivary metagenome with a mean number per sample of 11 phyla (10–12), 115 genera (95–123), and 321 species (240–351). Metatranscriptomic bacterial identification yielded a total number of 12 bacterial phyla, 101 bacterial genera, and 234 bacterial species with a mean number per sample of 10 phyla (8–12), 101 genera (79–120), and 262 species (204–324).

**Fig. 2:**
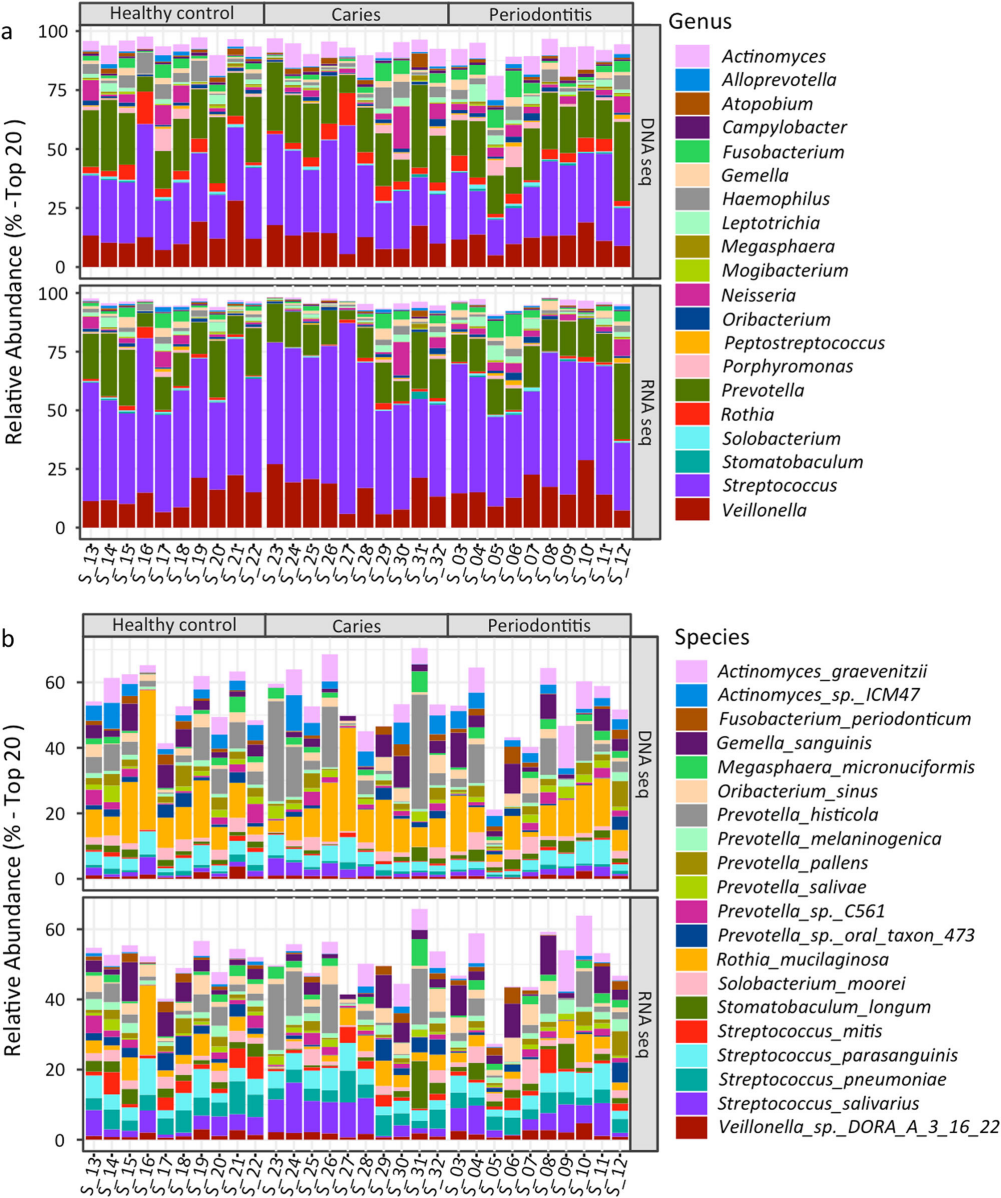
Relative abundance. **a**: Top 20 predominant microbial genera. **b**: Top 20 predominant microbial species based on metagenomic and metatranscriptomic analyses

Twenty most abundant bacterial genera based on metagenomic and metatranscriptomic bacterial identifications are displayed in Fig. 2a. As seen in Fig. 2a, the three most predominant genera based on both identification methods were *Streptococcus*, *Prevotella*, and *Veillonella* constituting approx. 70% of all assigned DNA and RNA reads. *Streptococcus* was significantly more abundant based on metatranscriptomic identification (50%) than metagenomic identification (25%, *p* < 0.05).

The predominant 20 bacterial species based on metagenomic and metatranscriptomic bacterial identifications are displayed in Fig. 2b. While the salivary metagenomes were dominated by equal proportions of *Streptococcus sp*. and *Rothia sp*. the salivary metatranscriptomes were characterized by high proportions of *Streptococcus sp*. but very low proportions of *Rothia sp*.

### Salivary abundance of specific oral bacterial species associates with periodontitis and dental caries

**Fig. 3:**
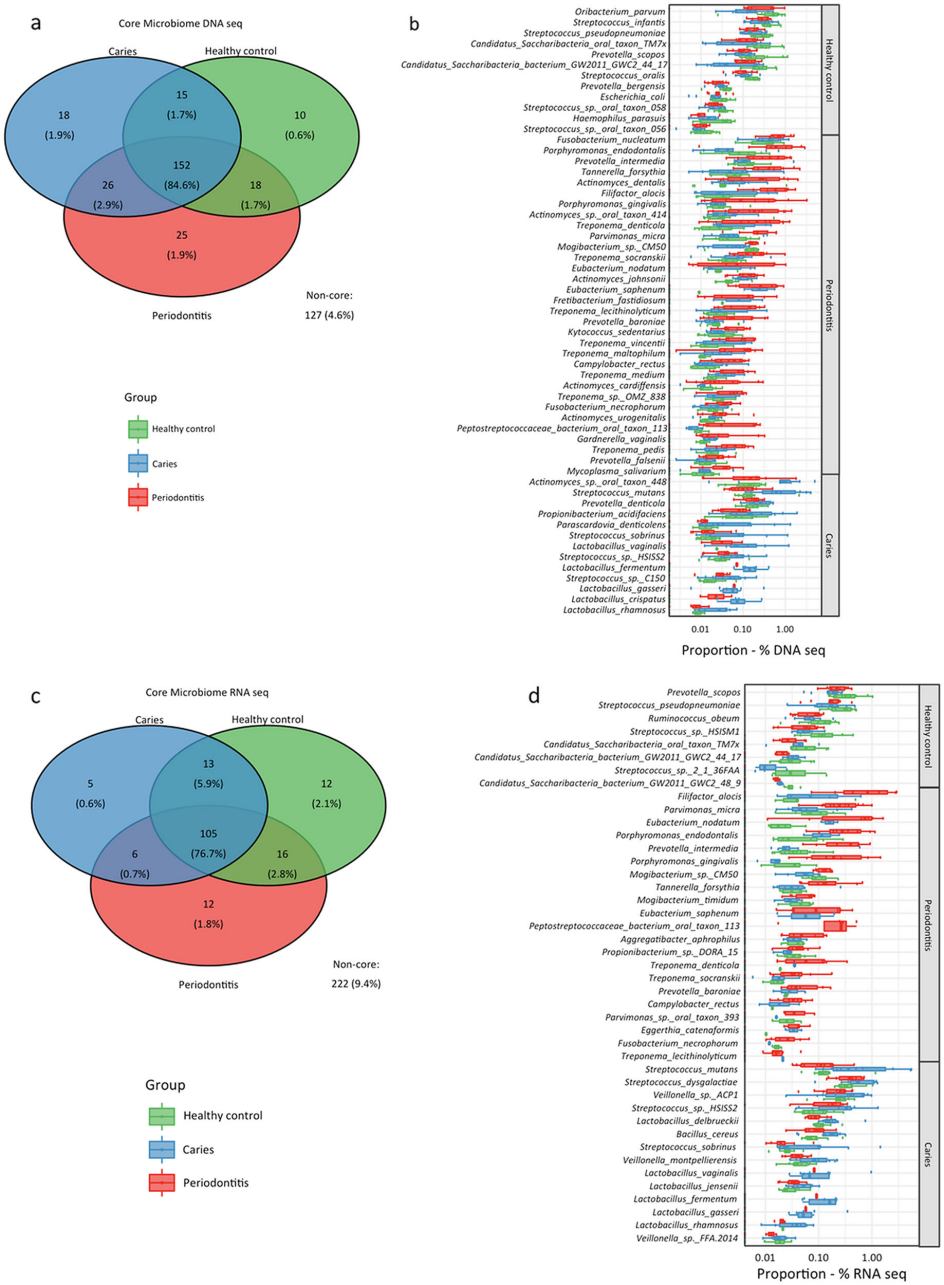
Periodontitis and caries-associated salivary microbiota. **a**: Venn diagram displaying number and proportion of microbial species in salivary core and non-core microbiome based on metagenomic approach. **b:** Bacterial species with significantly higher relative abundance in oral health, dental caries or periodontitis. **c**: Venn diagram displaying number and proportion of microbial species in salivary core and non-core microbiome based on metatranscriptomic approach. **d**: Bacterial species with significantly higher relative abundance in oral health, dental caries or periodontitis. Sample denotation: green: healthy controls, blue: dental caries, red: periodontitis

From a total of 391 bacterial species identified using metagenomic analysis, 264 constituted the core salivary microbiome, and the majority of bacterial species (84.9%) were shared between samples from periodontitis patients, dental caries patients, and orally healthy controls (Fig. 3a). In addition, 32 periodontitis-associated, 13 caries-associated, and 11 oral health-associated microbial species were identified based on relative abundance of assigned DNA reads (*p* < 0.05, Fig. 3b).

Based on metatranscriptomic analysis a total of 105 bacterial species constituting 76.7% of the total relative abundance were shared between the three groups (Fig. 3c). In addition, 22 periodontitis-associated, 14 caries-associated, and 8 oral health-associated microbial species were identified based on relative abundance of assigned RNA reads (*p* < 0.05, Fig. 3d).

Traditional periodontal pathogens such as *P. gingivalis*, *Tannerella forsythia*, *Parvimonas micra*, and *F. alocis* and cariogenic bacteria, including *S. mutans* and *Lactobacillus sp*. were identified with significantly higher relative abundance in samples from periodontitis and dental caries patients, respectively, based on both metagenomic and metatranscriptomic approaches (Figs. 3b, d).

### Microbial activity of specific oral bacterial species associates with oral health and disease

**Fig. 4:**
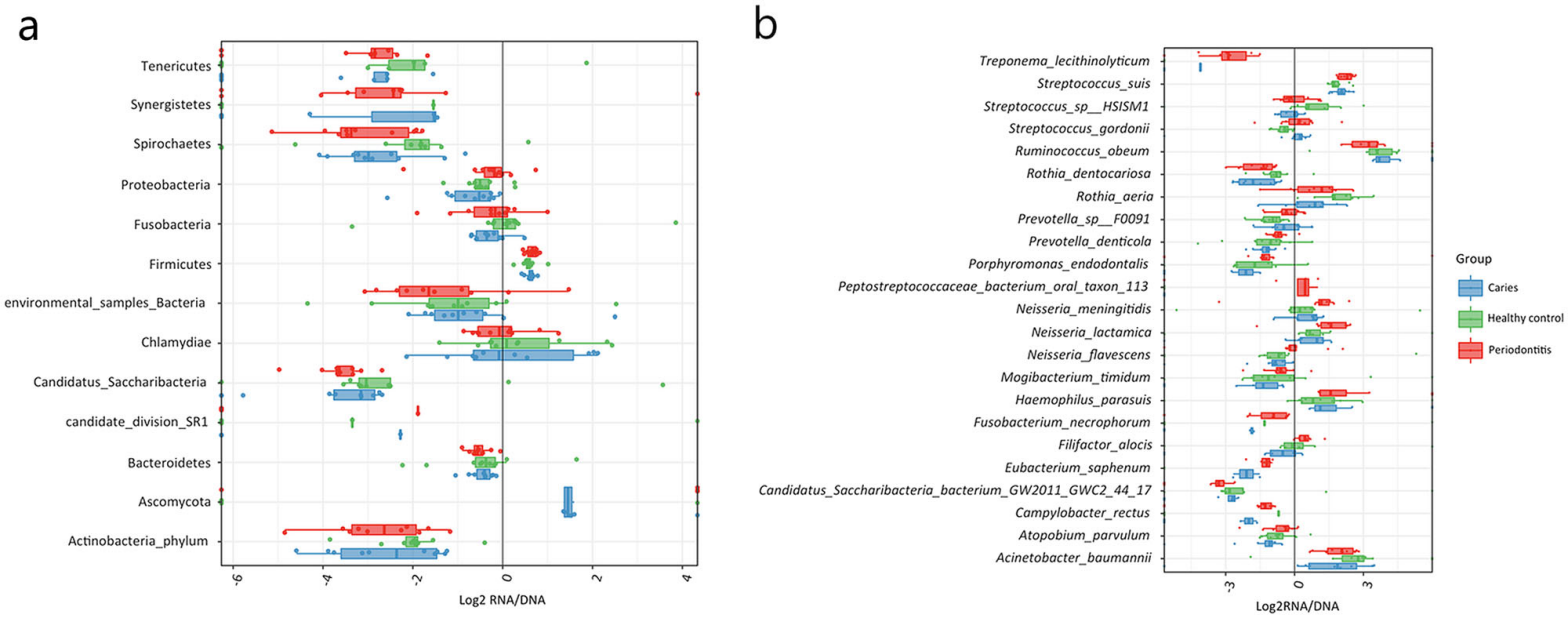
Salivary microbial activity. **a**: Log2 RNA/DNA ratio of the 13 bacterial phyla identified in saliva across all samples. **b**: Bacterial species identified with significantly different log2 RNA/DNA ratios in samples from patients with periodontitis (red), dental caries patients (blue) and orally healthy controls (green)

Based on comparison of RNA/DNA ratio at phylum level Firmicutes was the only taxon expressing a positive RNA/DNA ratio in all groups, while the majority of remaining 12 phyla were recorded with negative RNA/DNA ratios (Fig. 4a). A total of 23 bacterial species were identified with significantly different RNA/DNA ratios between groups (*p* < 0.05) (Fig. 4b).

### Salivary metabolic gene expression in periodontitis, caries, and oral health

**Fig. 5:**
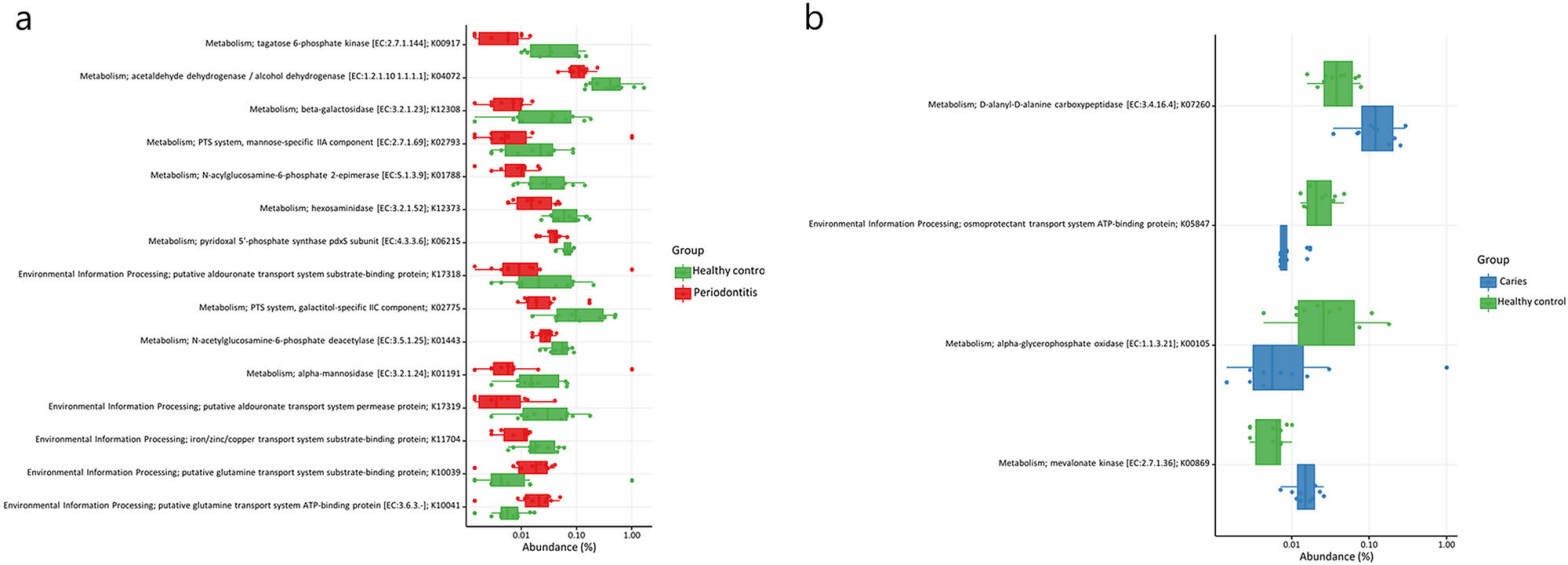
Differentially expressed genes in the salivary metatranscriptome in dental health and disease. **a**: periodontitis patients (red) and healthy controls (green) and **b**: dental caries patients (blue) and healthy controls (green). Only significant differences as determined using DESeq2 negative binomial tests are shown (*p* < 0.05). KEGG orthologs (KO) numbers and Enzyme commission numbers are reported (EC) when applicable

Differential gene expression analysis (i.e., metatranscriptomic data) allowed the identification of 15 differentially expressed KO between periodontitis patients and healthy controls (2 periodontitis-associated and 13 health-associated), out of which ten belonged to the KO metabolism category and five belonged to the KO Environmental Processing Information category (*p* < 0.05, Fig. 5a). The majority of KO, which was down-regulated in periodontitis compared to orally healthy controls, was involved in carbohydrate metabolism (K00917, K04072, K12308, K02793, K01788, K12373, K02775, and K01443). Four differentially expressed KO were recorded between caries patients and healthy controls (two caries-associated and two health-associated), from which three belonged to the KO metabolism category and one belonged to the KO environmental processing information category (*p* < 0.05; Fig. 5b). The two KO, which were up-regulated in caries was involved in glycan biosynthesis (K07260) and carbohydrate metabolism (K00869), whereas lipid metabolism (K00105) was up-regulated in orally healthy controls compared to caries patients (Fig. 5b).

## Discussion

The purpose of this study was to characterize and compare salivary microbiota in oral health and disease using metagenomic and metatranscriptomic approaches. The primary finding was that both metagenomic and metatranscriptomic analyses identified higher salivary abundance of specific oral bacterial species in periodontitis and dental caries patients. Furthermore, salivary expression of specific metabolic genes was significantly different in patients with periodontitis and caries compared to healthy controls, and high activity (RNA/DNA ratio) of *F. alocis* and *Neisseria sp*. was associated with periodontitis. To the best of our knowledge, this is the first study to perform comprehensive simultaneous metagenomic and metatranscriptomic characterization of the salivary microbiota.

*Streptococcus* was identified as the most predominant bacterial genus by use of both metagenomic and metatranscriptomic analyses (Figs. 2a–b). These findings are in line with 16S-metabarcoding and metaproteomic analyses performed on the same samples,^17,21^ and underline the importance of *Streptococcus* in the composition of the salivary microbiota. Moreover, metagenomic and metatranscriptomic analyses yielded significant differences in characterization of the salivary microbiota (Figs. 2a–b). Remarkably, this could be acknowledged at genus level where *Streptococcus* accounted for approx. 25% of all assigned DNA reads compared to 50% of all assigned RNA reads. This finding was further supported, as phylum Firmicutes, which includes the genus *Streptococcus*, was the most active bacterial phylum in saliva as determined by the RNA/DNA ratio (Fig. 4a). Interestingly, this observation aligns with metagenomic and metatranscriptomic data extracted from a batch of supragingival samples, in which major differences in bacterial metagenomic and metatranscriptomic profiles were observed.^5,8^ Thus, this observation suggests that some bacterial genera, including *Streptococcus* may constitute the more active part of the salivary microbiota, while other members of the salivary microbiota, for example *Rothia sp*. might be largely inactive.

Based on relative abundance of the taxa at metagenomic level, multiple periodontitis-associated and caries-associated bacterial species were identified (Fig. 3b). These findings are in line with previous studies employing various contemporary molecular methods for bacterial identification in saliva,^17,18,20,22–24^ including 16S-metabarcoding identification performed on the same saliva samples.^17^ Subgingival colonization of bacterial species such as *P. gingivalis*, *T. forsythia*, *P. micra*, and *F. alocis* is considered strongly associated with periodontal disease activity.^6,25,26^ Furthermore, *S. mutans* and *Lactobacillus sp*. are considered key etiological agents in the cariogenic supragingival biofilm based on their proficient capability of sugar fermentation.^7,27^ These specific and well-known disease-associated bacterial species were identified in higher relative abundance in saliva samples from patients with periodontitis and dental caries. Essentially, this finding reinforces the concept that pathogenic bacteria are shed from local periodontitis and caries lesions into saliva. This may increase salivary abundance of these bacteria in patients with periodontitis and dental caries. However, low levels of *P. gingivalis* and *S. mutans* are also occasionally identified in saliva from orally healthy individuals,^28,29^ indicating that in some individuals these bacteria may be part of the resident oral microbiota.

The salivary microbiota has been reported to primarily consist of bacteria shed from the tongue, throat and tonsils.^16^ The turn-over time of the salivary microbiota is extremely high due to continuous swallowing.^15^ Bacterial activity is ultimately determined by the level of substrates and local environmental conditions.^30,31^ Thus, bacterial activity of the salivary microbiota may relate to remnants of the phenotypic profile expressed in the local environment. Functional analysis of metatranscriptomic data highlighted significant differences in salivary gene expression in patients with periodontitis and caries compared to orally healthy controls. Saliva from orally healthy controls was associated with an up-regulation in functional activity of carbohydrate metabolism compared to periodontitis patients (Fig. 5a). Furthermore, saliva from caries patients expressed up-regulation of carbohydrate metabolism and down-regulation of lipid-metabolism compared to orally healthy controls (Fig. 5b). Thus, data from this study clearly demonstrate bacterial metabolic activity of the salivary microbiota at the community level, which is surprising, when considering the high turn-over rate of the salivary microbiota. Unfortunately, the sequencing depth was not sufficient for comparisons of species-specific metabolic activity between groups. However, as approx. 50% of all RNA reads could be taxonomically assigned to *Streptococcus* species it is likely that a substantial part of salivary metabolic activity derived from *Streptococcus* species, which is further supported by the fact that *Streptococcus* species are proficient in carbohydrate metabolism.^32^ Notably, 16S-metabarcoding analysis of the same samples showed significantly lower relative abundance of *Streptococcus* species in samples from periodontitis patients compared to healthy controls,^17^ which suggests that metatranscriptomic profiles observed in this study probably associates with differential functional activity of salivary *Streptococcus* species in oral health and disease.

Taxonomic assignment of metatranscriptomic data of the salivary microbiota was employed as a proxy marker of microbial activity. Notably, all the usual suspects of traditional periodontal pathogens (*P. gingivalis*, *T. forsythia*, *P. micra,* and *F. alocis*) and cariogenic bacteria (*S. mutans* and *Lactobacillus sp*.) were identified with significantly higher relative abundance at metatranscriptomic level (Fig. 3d). This suggests that bacteria shed from local diseased lesions sustain microbial activity when dispersed into saliva. This is further demonstrated by the higher salivary RNA/DNA ratio of *F. alocis* and *Neisseria sp*. in patients with periodontitis. Consequently, higher salivary bacterial activity of the usual suspects of periodontitis (*P. gingivalis*, *T. forsythia*, *P. micra*, and *F. alocis*) and dental caries (*S. mutans* and *Lactobacillus sp*.) may possess a potential for use as a biomarker of the respective diseases. Further studies with additional sequencing depth and larger sample size (including supra and subgingival samples) would allow the comparison of salivary and local diseased lesions’ microbiota. This would further strengthen the finding of the present study.

In conclusion, this study is the first to demonstrate differences in salivary metabolic gene expression in oral health and disease based on metatranscriptomic characterization of the salivary microbiota. Future longitudinal studies are warranted to reveal whether salivary screening of metabolic gene expression can identify oral diseases at preclinical stages.

## Methods

### Study population and sample collection

The study population and oral examination has been presented elsewhere in detail.^17^ In brief, 10 patients each, with chronic periodontitis, or manifest dental caries and orally healthy controls were enrolled in April 2015 at Copenhagen University, School of Dentistry. Periodontitis was defined as bleeding on probing ≥ 25% of total sites + minimum two teeth with clinical attachment level ≥ 4 mm + minimum two teeth with probing depth ≥ 6 mm and dental caries was defined as manifest untreated caries ≥ 3 surfaces.^17^ All 30 participants signed informed consent. The study was completed in accordance with all relevant guidelines and procedures, approved by the regional ethical committee (H-15000856) and reported to the Danish Dental Authorization (2015-54-0970).

Stimulated saliva samples (*n* = 30) were collected between 8:00 a.m. and 11:00 a.m. according to a standardized protocol.^33^ Immediately after collection each saliva sample was divided in four aliquots of 1 mL each. Two of these aliquots have previously been used for analysis of bacterial 16S metabarcoding targeting rRNA gene^17^ and metaproteomics.^21^ The two remaining aliquots were allocated for this study, one each for metagenomics and metatranscriptomics. RNAlater (Life Technologies, Denmark) was added to the aliquot allocated for metatranscriptomics and all aliquots were immediately stored at −80 °C until further processing.

### Metagenomic and metatranscriptomic library preparation and sequencing

Frozen saliva samples were thaw on ice and centrifuged to collect debris. Lysing Matrix B tube (MP Biomedicals) was used to lyse the debris (10 m, 30 s, twice). DNA and RNA (i.e., total RNA) were extracted using MasterPure^TM^ Complete DNA and RNA Purification Kit (Epientre, Chicago, IL, USA). For DNA samples, RNase was used to remove RNA contamination. For RNA samples, DNase was used to remove DNA contamination.

RNA quality, quantity and residual DNA were determined using a 2100 Bioanalyzer (Agilent Technologies, Santa Clara, CA, USA), as well as Invitrogen RiboGreen and PicoGreen assays (ThermoFisher Scientific, Waltham, MA, USA), respectively. Library preparation was performed according to the TruSeq Stranded mRNA protocol (Illumina, San Diego, CA, USA) with the following modifications: The oligo-dT mRNA purification step was omitted and instead, 200ng of total RNA were directly added to the Elution2-Frag-Prime step. The PCR amplification step, which selectively enriches for library fragments that have adapters ligated on both ends, was performed according to the manufacturer’s recommendation but the number of amplification cycles was reduced to 12. Each library was uniquely tagged with one of Illumina’s TruSeq LT RNA barcodes to enable library pooling for sequencing. RNA libraries were then sequenced in six lanes of Illumina Hiseq2500 rapid runs at a read-length of 100 bp paired-end, generating an average of 32.71 M reads per sample (26.40–38.44 M).

DNA quality and quantity were determined using a 2100 Bioanalyzer and Invitrogen’s PicoGreen assay, respectively. Library preparation was performed according to Illumina’s TruSeq Nano DNA Sample Preparation protocol. The samples were sheared on a Covaris S220 (Covaris, Woburn, MA, USA) to ~ 450 bp, following the manufacturer’s recommendation, and uniquely tagged with one of Illumina’s TruSeq LT DNA barcodes. DNA libraries were then sequenced in 2 lanes of an Illumina Hiseq2500 rapid run at a read-length of 250 bp paired-end, generating an average of 25.62 M (16.46–32.64 M) reads per sample.

Sequence data was deposited in GenBank (Sequence Read Archive) and is available under the BioProject (PRJNA396840).

### Read preprocessing

Illumina adaptors, 5′ or 3′ bases with quality scores lower than 20, as well as read pairs having a mate with any ambiguous base (i.e., N) or shorter than 150 or 50 bp for DNA and RNA sequences, respectively, were trimmed using cutadapt in paired-end mode (version 1.10, -a -A --max-n 0 -n 1 -q 20,20 --quality-base 33 --minimum-length 150/50 -O 6). Overall, quality-trimmed reads represented 98.1% (97.5−98.6%) of DNA and 99.3% (99.0−99.4%) of RNA reads.

RNA reads were then subjected to sortmeRNA (version 2.0),^34^ using provided rRNA databases and default parameters for *in silico* depletion of ribosomal RNA. Ribosomal RNA sequences represented an average of 83.9% of the quality-trimmed RNA reads (71.1−88.4%). Human reads were then removed from metagenomic and metatranscriptomic dataset by aligning DNA and mRNA reads to the human genome (h38, ftp://ftp.ncbi.nlm.nih.gov/genomes/all/GCF/000/001/405/GCF_000001405.36_GRCh38.p10/GCF_000001405.36_GRCh38.p10_genomic.fna.gz) using Bowtie2 (version 2.2.9, --un --sensitive-local). Overall, around 9.1 M (2.3–9.2 M) DNA and 4.9 M (2.9–9.1 M) RNA reads were used after quality filtering, rRNA removal and host (i.e., human) contamination for further analysis. Prior to further analysis, 2.26 M reads were randomly selected per sample to obtain equivalent sequencing depth and allow robust comparison.

### Taxonomic and functional community profiling

In order to characterize the taxonomic composition of metagenomic and metatranscriptomic dataset we used a similar approach as Ilott et al. 2016.^35^ Subsampled sequences were aligned against the NCBI non-redundant (NR) protein database (ftp.ncbi.nlm.nih.gov/blast/db/FASTA/nr.gz, March 2016) using DIAMOND (version 0.7.10.59)^36^ with default parameters. The lowest common ancestor approach implemented in MEGAN6 (L.C.A., version CE_6_5_5),^37^ -ms 50 (minimum alignment bitscore = 50) -supp 0 –sup 25) was used to assign reads at the phylum, genus and species levels for the metagenomic and metatranscriptomic dataset, respectively. In order to characterize the molecular level function of the salivary microbiome, aligned reads were also assigned to KO functional orthologs using GI to KEGG KO functional identifier mapping file as implemented in MEGAN6.^37^

### Statistical analysis

Taxonomic and functional count tables from metagenomic and metatranscriptomic dataset were exported from MEGAN6 and imported as single phyloseq objects (phyloseq, R). Taxonomic and functional tables were filtered to remove taxa accounting less than 100 sequences in total and observed in less than 3 samples (*filter_taxa* function). Tables were then rarefied to an even sequencing depth using *rarefy_even_depth* function.

Microbial communities were characterized using alpha-diversity indices (i.e., number of observed taxa and Shannon diversity indices) and beta-diversity (i.e., Bray–Curtis dissimilarity) at the Phylum, Genus and Species levels on both metagenomic and metatranscriptomic datasets.

Differences in alpha and beta diversity between healthy control, caries and periodontitis groups dataset were tested using ANOVA and PERMANOVA statistical tests for alpha and beta-diversity, respectively (i.e., *Adonis*, *PERMANOVA* functions). Gaussian distribution of ANOVA residuals and multivariate spread was tested to ensure ANOVA and PERMANOVA assumptions (i.e., *shapiro.test* and *betadisper)*. Statistical significance of taxa-group association was tested using one-sided signassoc function from the indicspecies R-package. Sidak’s correction was applied for multiple testing. RNA/DNA log ratios were determined for the relative abundance of taxa identified in metagenomic and metatranscriptomic dataset at phylum and species level. Due to skewed distribution of RNA/DNA log ratios, non-parametric Kruskal–Wallis *H* test was applied to test differences between groups.

Differential expression analysis of identified KO functional orthologs between healthy controls and patients with periodontitis and caries were conducted on unrarefied functional counts using DESeq2 standard meta-/transcriptomic pipeline which includes size factor normalization and standardization procedures as well as negative binomial model testing. pvalues were adjusted for multiple testing using FDR correction^38^—using the following parameters: test = “wald” and fitType = “parametric”).

### Data availability statement

Sequence data that support the findings of this study have been deposited in GenBank (Sequence Read Archive) and is available under the BioProject (PRJNA396840).

## Electronic supplementary material

Table S1

Figure S1

Figure S2
