# Supplementary material for: Metagenomic and metatranscriptomic analysis of saliva reveals disease-associated microbiota in patients with periodontitis and dental caries
Source: NPJ Biofilms Microbiomes. 2017 Oct 2;3:23. doi: 10.1038/s41522-017-0031-4 (PMC5624903; doi:10.1038/s41522-017-0031-4)
Supplement: Supplementary file 1 — Table S1 [file 41522_2017_31_MOESM1_ESM.docx]

| **File Name** | **Raw reads** | **Trimmed reads** | |  |  | **Non-human reads** | | **Subsampled reads to an even depth** | **Diamond Final Matches** | **Diamond Queries Aligned** | | **Megan Total Reads** | | **Reads With Hits** | | **Alignment** | **Assig. Taxonomy** | | **Assig. KEGG Ortholgos KO** | | **Class. Taxonomy** | **Reads assigned at the phylum level** | | **Reads assigned at the genus level** | | **Reads assigned at the species level** | |
| --- | --- | --- | --- | --- | --- | --- | --- | --- | --- | --- | --- | --- | --- | --- | --- | --- | --- | --- | --- | --- | --- | --- | --- | --- | --- | --- | --- |
| 4012DNA | 19,604,046 | 19,134,988 | 97.6% |  |  | 7,286,837 | 38.1% | 2,262,000 | 42,020,656 | 1,942,401 | 85.9% | 1,942,401 | 85.9% | 1,941,504 | 86% | 42,020,656 | 1,941,504 | 86% | 318,942 | 14% | 1,097 | 1,474,710 | 76% | 1,088,750 | 56.1% | 337,710 | 17% |
| 4014DNA | 28,664,578 | 27,982,304 | 97.6% |  |  | 14,536,605 | 51.9% | 2,262,000 | 41,047,726 | 1,944,278 | 86.0% | 1,944,278 | 86.0% | 1,940,847 | 86% | 41,047,726 | 1,940,846 | 86% | 409,422 | 18% | 1,086 | 1,471,050 | 76% | 1,151,564 | 59.3% | 362,471 | 19% |
| 4020DNA | 29,124,674 | 28,431,520 | 97.6% |  |  | 4,724,158 | 16.6% | 2,262,000 | 37,446,957 | 1,811,547 | 80.1% | 1,811,547 | 80.1% | 1,808,843 | 80% | 37,446,957 | 1,808,843 | 80% | 610,740 | 27% | 1,306 | 1,441,609 | 80% | 1,145,198 | 63.3% | 402,053 | 22% |
| 4024DNA | 27,479,450 | 26,847,696 | 97.7% |  |  | 11,248,453 | 41.9% | 2,262,000 | 40,795,865 | 1,925,912 | 85.1% | 1,925,912 | 85.1% | 1,924,848 | 85% | 40,795,865 | 1,924,847 | 85% | 436,566 | 19% | 1,168 | 1,452,487 | 75% | 1,067,370 | 55.5% | 339,640 | 18% |
| 4028DNA | 24,270,832 | 23,705,018 | 97.7% |  |  | 4,815,107 | 20.3% | 2,262,000 | 38,799,371 | 1,845,255 | 81.6% | 1,845,255 | 81.6% | 1,842,815 | 81% | 38,799,371 | 1,842,813 | 81% | 368,706 | 16% | 1,222 | 1,457,154 | 79% | 1,185,777 | 64.3% | 357,786 | 19% |
| 4032DNA | 23,701,070 | 23,300,642 | 98.3% |  |  | 6,282,373 | 27.0% | 2,262,000 | 43,497,572 | 1,971,653 | 87.2% | 1,971,653 | 87.2% | 1,970,722 | 87% | 43,497,572 | 1,970,721 | 87% | 631,098 | 28% | 1,019 | 1,481,316 | 75% | 1,114,224 | 56.5% | 302,064 | 15% |
| 4036DNA | 20,327,982 | 20,014,618 | 98.5% |  |  | 6,336,055 | 31.7% | 2,262,000 | 43,350,366 | 1,993,942 | 88.1% | 1,993,942 | 88.1% | 1,993,541 | 88% | 43,350,366 | 1,993,541 | 88% | 540,618 | 24% | 954 | 1,488,016 | 75% | 1,196,897 | 60.0% | 316,666 | 16% |
| 4040DNA | 26,411,014 | 26,006,224 | 98.5% |  |  | 4,988,468 | 19.2% | 2,262,000 | 41,552,848 | 1,929,560 | 85.3% | 1,929,560 | 85.3% | 1,928,593 | 85% | 41,552,848 | 1,928,593 | 85% | 411,684 | 18% | 999 | 1,469,588 | 76% | 1,188,470 | 61.6% | 345,028 | 18% |
| 4044DNA | 24,724,732 | 24,372,006 | 98.6% |  |  | 8,061,661 | 33.1% | 2,262,000 | 42,098,416 | 1,932,049 | 85.4% | 1,932,049 | 85.4% | 1,929,921 | 85% | 42,098,416 | 1,929,921 | 85% | 389,064 | 17% | 1,163 | 1,461,051 | 76% | 1,172,040 | 60.7% | 338,001 | 18% |
| 4048DNA | 25,454,656 | 25,063,454 | 98.5% |  |  | 13,766,745 | 54.9% | 2,262,000 | 42,832,179 | 1,981,536 | 87.6% | 1,981,536 | 87.6% | 1,979,852 | 88% | 42,832,179 | 1,979,852 | 88% | 285,012 | 13% | 1,112 | 1,474,545 | 74% | 1,126,160 | 56.9% | 288,162 | 15% |
| 4050DNA | 25,570,026 | 24,981,546 | 97.7% |  |  | 11,566,964 | 46.3% | 2,262,000 | 41,938,022 | 1,932,194 | 85.4% | 1,932,194 | 85.4% | 1,929,143 | 85% | 41,938,022 | 1,929,143 | 85% | 445,614 | 20% | 1,041 | 1,476,750 | 77% | 1,141,578 | 59.2% | 281,615 | 15% |
| 4056DNA | 32,637,106 | 31,862,162 | 97.6% |  |  | 17,735,623 | 55.7% | 2,262,000 | 41,050,079 | 1,931,682 | 85.4% | 1,931,682 | 85.4% | 1,920,966 | 85% | 41,050,079 | 1,920,966 | 85% | 427,518 | 19% | 1,108 | 1,445,930 | 75% | 1,098,309 | 57.2% | 340,124 | 18% |
| 4060DNA | 24,847,322 | 24,272,684 | 97.7% |  |  | 12,493,033 | 51.5% | 2,262,000 | 42,397,349 | 1,962,070 | 86.7% | 1,962,070 | 86.7% | 1,960,550 | 87% | 42,397,349 | 1,960,550 | 87% | 475,020 | 21% | 979 | 1,478,458 | 75% | 1,066,171 | 54.4% | 298,699 | 15% |
| 4064DNA | 21,851,468 | 21,343,406 | 97.7% |  |  | 5,150,238 | 24.1% | 2,262,000 | 43,612,179 | 1,925,857 | 85.1% | 1,925,857 | 85.1% | 1,925,563 | 85% | 43,612,179 | 1,925,563 | 85% | 416,208 | 18% | 825 | 1,494,017 | 78% | 1,096,315 | 56.9% | 275,064 | 14% |
| 4068DNA | 30,275,720 | 29,562,478 | 97.6% |  |  | 18,273,058 | 61.8% | 2,262,000 | 40,709,537 | 1,906,681 | 84.3% | 1,906,681 | 84.3% | 1,904,531 | 84% | 40,709,537 | 1,904,530 | 84% | 517,998 | 23% | 1,121 | 1,472,892 | 77% | 1,061,812 | 55.8% | 296,509 | 16% |
| 4072DNA | 25,623,136 | 25,222,688 | 98.4% |  |  | 12,053,063 | 47.8% | 2,262,000 | 42,339,600 | 1,956,612 | 86.5% | 1,956,612 | 86.5% | 1,954,028 | 86% | 42,339,600 | 1,954,028 | 86% | 420,732 | 19% | 1,090 | 1,466,638 | 75% | 1,092,482 | 55.9% | 302,874 | 15% |
| 4074DNA | 19,598,488 | 19,319,958 | 98.6% |  |  | 7,869,723 | 40.7% | 2,262,000 | 43,142,200 | 1,952,882 | 86.3% | 1,952,882 | 86.3% | 1,951,844 | 86% | 43,142,200 | 1,951,844 | 86% | 332,514 | 15% | 910 | 1,478,641 | 76% | 1,065,484 | 54.6% | 268,123 | 14% |
| 4080DNA | 30,473,660 | 29,993,170 | 98.4% |  |  | 18,516,964 | 61.7% | 2,262,000 | 41,494,224 | 1,965,038 | 86.9% | 1,965,038 | 86.9% | 1,960,838 | 87% | 41,494,224 | 1,960,838 | 87% | 404,898 | 18% | 1,122 | 1,461,354 | 75% | 1,142,337 | 58.3% | 360,646 | 18% |
| 4082DNA | 27,391,440 | 27,007,272 | 98.6% |  |  | 11,575,980 | 42.9% | 2,262,000 | 42,368,788 | 1,935,541 | 85.6% | 1,935,541 | 85.6% | 1,931,028 | 85% | 42,368,788 | 1,931,028 | 85% | 538,356 | 24% | 955 | 1,468,948 | 76% | 1,163,861 | 60.3% | 287,846 | 15% |
| 4088DNA | 23,664,574 | 23,293,812 | 98.4% |  |  | 4,403,091 | 18.9% | 2,262,000 | 41,485,863 | 1,923,809 | 85.0% | 1,923,809 | 85.0% | 1,914,412 | 85% | 41,485,863 | 1,914,412 | 85% | 300,846 | 13% | 1,124 | 1,460,695 | 76% | 1,182,644 | 61.8% | 325,673 | 17% |
| 4092DNA | 22,497,730 | 21,940,374 | 97.5% |  |  | 7,031,495 | 32.0% | 2,262,000 | 41,996,776 | 1,906,056 | 84.3% | 1,906,056 | 84.3% | 1,905,731 | 84% | 41,996,776 | 1,905,730 | 84% | 296,322 | 13% | 948 | 1,485,539 | 78% | 1,306,117 | 68.5% | 330,674 | 17% |
| 4096DNA | 29,014,792 | 28,341,726 | 97.7% |  |  | 9,350,142 | 33.0% | 2,262,000 | 42,749,283 | 1,949,887 | 86.2% | 1,949,887 | 86.2% | 1,947,285 | 86% | 42,749,283 | 1,947,284 | 86% | 343,824 | 15% | 1,018 | 1,478,098 | 76% | 1,172,149 | 60.2% | 331,496 | 17% |
| 4100DNA | 22,763,886 | 22,241,074 | 97.7% |  |  | 5,580,221 | 25.1% | 2,262,000 | 40,406,327 | 1,889,145 | 83.5% | 1,889,145 | 83.5% | 1,886,853 | 83% | 40,406,327 | 1,886,853 | 83% | 350,610 | 16% | 1,176 | 1,469,024 | 78% | 1,163,154 | 61.6% | 334,374 | 18% |
| 4104DNA | 25,888,904 | 25,288,238 | 97.7% |  |  | 11,390,217 | 45.0% | 2,262,000 | 42,981,963 | 1,961,591 | 86.7% | 1,961,591 | 86.7% | 1,961,432 | 87% | 42,981,963 | 1,961,432 | 87% | 581,334 | 26% | 941 | 1,484,344 | 76% | 1,196,389 | 61.0% | 354,199 | 18% |
| 4108DNA | 23,968,634 | 23,397,818 | 97.6% |  |  | 2,580,301 | 11.0% | 2,262,000 | 39,671,049 | 1,770,707 | 78.3% | 1,770,707 | 78.3% | 1,770,313 | 78% | 39,671,049 | 1,770,313 | 78% | 334,776 | 15% | 1,134 | 1,483,209 | 84% | 1,165,989 | 65.9% | 338,452 | 19% |
| 4116DNA | 31,792,524 | 31,279,704 | 98.4% |  |  | 5,349,407 | 17.1% | 2,262,000 | 41,231,583 | 1,917,106 | 84.8% | 1,917,106 | 84.8% | 1,916,222 | 85% | 41,231,583 | 1,916,222 | 85% | 282,750 | 13% | 1,202 | 1,468,593 | 77% | 1,187,942 | 62.0% | 375,242 | 20% |
| 4120DNA | 16,461,078 | 16,212,356 | 98.5% |  |  | 5,574,319 | 34.4% | 2,262,000 | 43,047,269 | 1,988,568 | 87.9% | 1,988,568 | 87.9% | 1,988,018 | 88% | 43,047,269 | 1,988,018 | 88% | 269,178 | 12% | 1,056 | 1,490,653 | 75% | 1,117,524 | 56.2% | 318,911 | 16% |
| 4124DNA | 29,306,412 | 28,861,028 | 98.5% |  |  | 4,569,519 | 15.8% | 2,262,000 | 43,261,070 | 1,959,818 | 86.6% | 1,959,818 | 86.6% | 1,957,136 | 87% | 43,261,070 | 1,957,136 | 87% | 246,558 | 11% | 995 | 1,479,162 | 76% | 1,068,895 | 54.6% | 280,236 | 14% |
| 4128DNA | 22,779,686 | 22,416,726 | 98.4% |  |  | 10,866,093 | 48.5% | 2,262,000 | 42,712,917 | 1,986,014 | 87.8% | 1,986,014 | 87.8% | 1,985,732 | 88% | 42,712,917 | 1,985,732 | 88% | 323,466 | 14% | 886 | 1,481,274 | 75% | 1,153,863 | 58.1% | 361,235 | 18% |
| 4132DNA | 32,447,250 | 31,955,010 | 98.5% |  |  | 10,198,426 | 31.9% | 2,262,000 | 42,200,836 | 1,963,946 | 86.8% | 1,963,946 | 86.8% | 1,958,617 | 87% | 42,200,836 | 1,958,616 | 87% | 635,622 | 28% | 1,108 | 1,466,846 | 75% | 1,067,747 | 54.5% | 328,749 | 17% |
|  |  |  |  |  |  |  |  |  |  |  |  |  |  |  |  |  |  |  |  |  |  |  |  |  |  |  |  |
| min | 16,461,078 | 16,212,356 | 97.5% |  |  | 2,580,301 | 11.0% | 2,262,000 | 37,446,957 | 1,770,707 | 78.3% | 1,770,707 | 78.3% | 1,770,313 | 78.3% | 37,446,957 | 1,770,313 | 78.3% | 246,558 | 10.9% | 825 | 1,441,609 | 74.5% | 1,061,812 | 54.4% | 268,123 | 13.7% |
| max | 32,637,106 | 31,955,010 | 98.6% |  |  | 18,516,964 | 61.8% | 2,262,000 | 43,612,179 | 1,993,942 | 88.1% | 1,993,942 | 88.1% | 1,993,541 | 88.1% | 43,612,179 | 1,993,541 | 88.1% | 635,622 | 28.1% | 1,306 | 1,494,017 | 83.8% | 1,306,117 | 68.5% | 402,053 | 22.2% |
| average | 25,620,562 | 25,121,723 | 98.1% |  |  | 9,139,145 | 36.0% | 2,262,000 | 41,807,962 | 1,932,111 | 85.4% | 1,932,111 | 85.4% | 1,929,724 | 85.3% | 41,807,962 | 1,929,724 | 85.3% | 411,533 | 18.2% | 1,062 | 1,472,086 | 76.3% | 1,138,240 | 59.0% | 326,011 | 16.9% |
| stdev | 4,030,673 | 3,949,061 | 0.4% |  |  | 4,414,377 | 14.6% | - | 1,413,160 | 49,775 | 2.2% | 49,775 | 2.2% | 49,871 | 2.2% | 1,413,160 | 49,871 | 2.2% | 111,719 | 5% | 109 | 12,583 | 1.9% | 55,474 | 3.6% | 32,619 | 1.9% |
| sum | 768,616,870 | 753,651,700 |  |  |  | 274,174,339 |  | 67,860,000 | 1,254,238,870 | 57,963,337 |  | 57,963,337 |  | 57,891,728 |  | 1,254,238,870 | 57,891,719 |  |  |  |  |  |  |  |  |  |  |
|  |  |  |  |  |  |  |  |  |  |  |  |  |  |  |  |  |  |  |  |  |  |  |  |  |  |  |  |
| **File Name** | **Raw reads** | **Trimmed reads** | | **mRNA Reads** | | **Non-human reads** | | **Subsampled reads to an even depth** | **Diamond Final Matches** | **Diamond Queries Aligned** | | **Megan Total Reads** | | **Reads With Hits** | | **Alignment** | **Assig. Taxonomy** | | **Assig. KEGG Ortholgos KO** | | **Class. Taxonomy** | **Reads assigned at the phylum level** | | **Reads assigned at the genus level** | | **Reads assigned at the species level** | |
| 4012RNA | 32,952,773 | 32,722,949 | 99.3% | 5,448,442 | 16.7% | 4,194,453 | 77.0% | 2,262,000 | 37,274,873 | 1,650,165 | 73.0% | 1,650,165 | 73.0% | 1,648,591 | 73% | 37,274,873 | 1,648,591 | 73% | 1,210,170 | 54% | 769 | 1,444,390 | 88% | 1,123,857 | 68.2% | 171,075 | 10% |
| 4016RNA | 30,951,971 | 30,695,282 | 99.2% | 3,645,056 | 11.9% | 3,557,405 | 97.6% | 2,262,000 | 37,181,089 | 1,656,818 | 73.2% | 1,656,818 | 73.2% | 1,654,396 | 73% | 37,181,089 | 1,654,396 | 73% | 1,239,576 | 55% | 699 | 1,432,765 | 87% | 1,112,144 | 67.2% | 191,427 | 12% |
| 4020RNA | 35,915,016 | 35,665,942 | 99.3% | 6,123,196 | 17.2% | 5,684,496 | 92.8% | 2,262,000 | 31,688,176 | 1,577,935 | 69.8% | 1,577,935 | 69.8% | 1,575,837 | 70% | 31,688,176 | 1,575,837 | 70% | 1,194,336 | 53% | 956 | 1,413,524 | 90% | 1,053,203 | 66.8% | 222,063 | 14% |
| 4024RNA | 33,583,684 | 33,366,577 | 99.4% | 6,357,060 | 19.1% | 6,103,518 | 96.0% | 2,262,000 | 32,048,092 | 1,574,595 | 69.6% | 1,574,595 | 69.6% | 1,572,983 | 70% | 32,048,092 | 1,572,983 | 70% | 1,004,328 | 44% | 809 | 1,413,144 | 90% | 956,765 | 60.8% | 228,506 | 15% |
| 4028RNA | 30,380,467 | 30,180,987 | 99.3% | 4,400,522 | 14.6% | 4,084,880 | 92.8% | 2,262,000 | 33,346,673 | 1,644,349 | 72.7% | 1,644,349 | 72.7% | 1,642,666 | 73% | 33,346,673 | 1,642,666 | 73% | 1,164,930 | 52% | 841 | 1,427,246 | 87% | 1,130,249 | 68.8% | 206,117 | 13% |
| 4032RNA | 33,546,167 | 33,194,581 | 99.0% | 9,625,062 | 29.0% | 9,108,755 | 94.6% | 2,262,000 | 40,342,742 | 1,699,115 | 75.1% | 1,699,115 | 75.1% | 1,697,231 | 75% | 40,342,742 | 1,697,231 | 75% | 1,090,284 | 48% | 592 | 1,461,559 | 86% | 1,101,108 | 64.9% | 164,869 | 10% |
| 4036RNA | 36,878,879 | 36,576,636 | 99.2% | 5,270,184 | 14.4% | 5,160,368 | 97.9% | 2,262,000 | 40,031,549 | 1,706,089 | 75.4% | 1,706,089 | 75.4% | 1,703,616 | 75% | 40,031,549 | 1,703,614 | 75% | 1,099,332 | 49% | 665 | 1,455,636 | 85% | 1,161,784 | 68.2% | 163,523 | 10% |
| 4040RNA | 35,715,912 | 35,488,638 | 99.4% | 5,390,348 | 15.2% | 5,193,696 | 96.4% | 2,262,000 | 36,198,912 | 1,704,643 | 75.4% | 1,704,643 | 75.4% | 1,703,096 | 75% | 36,198,912 | 1,703,096 | 75% | 1,257,672 | 56% | 714 | 1,429,703 | 84% | 1,161,854 | 68.2% | 185,364 | 11% |
| 4044RNA | 38,093,322 | 37,802,140 | 99.2% | 5,222,146 | 13.8% | 5,056,664 | 96.8% | 2,262,000 | 35,369,797 | 1,616,971 | 71.5% | 1,616,971 | 71.5% | 1,615,370 | 71% | 35,369,797 | 1,615,370 | 71% | 1,035,996 | 46% | 796 | 1,429,536 | 88% | 1,098,552 | 68.0% | 199,419 | 12% |
| 4048RNA | 34,936,486 | 34,673,457 | 99.2% | 4,853,030 | 14.0% | 4,662,272 | 96.1% | 2,262,000 | 31,632,200 | 1,586,573 | 70.1% | 1,586,573 | 70.1% | 1,584,765 | 70% | 31,632,200 | 1,584,765 | 70% | 1,112,904 | 49% | 891 | 1,425,639 | 90% | 1,032,752 | 65.2% | 216,606 | 14% |
| 4052RNA | 32,974,042 | 32,726,883 | 99.3% | 5,158,150 | 15.8% | 5,034,769 | 97.6% | 2,262,000 | 37,579,577 | 1,724,758 | 76.2% | 1,724,758 | 76.2% | 1,722,048 | 76% | 37,579,577 | 1,722,048 | 76% | 1,230,528 | 54% | 750 | 1,448,645 | 84% | 1,116,042 | 64.8% | 158,684 | 9% |
| 4056RNA | 35,073,794 | 34,841,815 | 99.3% | 5,347,120 | 15.3% | 5,194,122 | 97.1% | 2,262,000 | 32,111,389 | 1,580,648 | 69.9% | 1,580,648 | 69.9% | 1,577,656 | 70% | 32,111,389 | 1,577,656 | 70% | 1,133,262 | 50% | 775 | 1,419,312 | 90% | 1,060,624 | 67.2% | 211,270 | 13% |
| 4060RNA | 34,046,538 | 33,856,568 | 99.4% | 5,426,728 | 16.0% | 5,240,478 | 96.6% | 2,262,000 | 33,721,267 | 1,632,374 | 72.2% | 1,632,374 | 72.2% | 1,630,691 | 72% | 33,721,267 | 1,630,691 | 72% | 1,314,222 | 58% | 750 | 1,432,701 | 88% | 1,014,879 | 62.2% | 201,317 | 12% |
| 4064RNA | 32,582,255 | 32,373,705 | 99.4% | 4,034,202 | 12.5% | 3,910,632 | 96.9% | 2,262,000 | 38,333,010 | 1,630,434 | 72.1% | 1,630,434 | 72.1% | 1,628,724 | 72% | 38,333,010 | 1,628,724 | 72% | 925,158 | 41% | 616 | 1,446,931 | 89% | 1,102,769 | 67.7% | 169,038 | 10% |
| 4068RNA | 28,091,643 | 27,929,674 | 99.4% | 3,801,614 | 13.6% | 3,685,251 | 96.9% | 2,262,000 | 31,598,446 | 1,539,579 | 68.1% | 1,539,579 | 68.1% | 1,537,919 | 68% | 31,598,446 | 1,537,919 | 68% | 1,244,100 | 55% | 894 | 1,426,429 | 93% | 959,326 | 62.4% | 210,419 | 14% |
| 4072RNA | 26,783,796 | 26,525,412 | 99.0% | 4,368,280 | 16.5% | 4,222,859 | 96.7% | 2,262,000 | 32,944,679 | 1,584,904 | 70.1% | 1,584,904 | 70.1% | 1,583,157 | 70% | 32,944,679 | 1,583,157 | 70% | 1,318,746 | 58% | 835 | 1,421,672 | 90% | 1,003,233 | 63.4% | 206,063 | 13% |
| 4076RNA | 26,400,683 | 26,191,989 | 99.2% | 3,992,796 | 15.2% | 3,881,254 | 97.2% | 2,262,000 | 36,394,263 | 1,620,947 | 71.7% | 1,620,947 | 71.7% | 1,619,539 | 72% | 36,394,263 | 1,619,539 | 72% | 1,280,292 | 57% | 635 | 1,438,905 | 89% | 1,056,257 | 65.2% | 162,784 | 10% |
| 4080RNA | 38,443,941 | 38,122,679 | 99.2% | 4,463,732 | 11.7% | 4,292,394 | 96.2% | 2,262,000 | 30,684,240 | 1,541,594 | 68.2% | 1,541,594 | 68.2% | 1,539,411 | 68% | 30,684,240 | 1,539,411 | 68% | 1,054,092 | 47% | 860 | 1,420,457 | 92% | 1,092,608 | 71.0% | 225,427 | 15% |
| 4084RNA | 34,572,356 | 34,311,044 | 99.2% | 5,121,050 | 14.9% | 4,748,377 | 92.7% | 2,262,000 | 35,091,758 | 1,599,524 | 70.7% | 1,599,524 | 70.7% | 1,596,709 | 71% | 35,091,758 | 1,596,709 | 71% | 1,153,620 | 51% | 642 | 1,433,678 | 90% | 1,113,640 | 69.7% | 187,205 | 12% |
| 4088RNA | 28,877,902 | 28,629,548 | 99.1% | 4,974,586 | 17.4% | 4,667,504 | 93.8% | 2,262,000 | 35,479,414 | 1,671,898 | 73.9% | 1,671,898 | 73.9% | 1,668,968 | 74% | 35,479,414 | 1,668,968 | 74% | 1,164,930 | 52% | 784 | 1,437,900 | 86% | 1,110,514 | 66.5% | 206,045 | 12% |
| 4092RNA | 32,039,879 | 31,849,555 | 99.4% | 5,750,702 | 18.1% | 5,550,396 | 96.5% | 2,262,000 | 38,303,728 | 1,684,742 | 74.5% | 1,684,742 | 74.5% | 1,683,551 | 74% | 38,303,728 | 1,683,551 | 74% | 1,207,908 | 53% | 567 | 1,455,517 | 86% | 1,245,282 | 74.0% | 169,111 | 10% |
| 4096RNA | 34,257,953 | 34,066,747 | 99.4% | 7,199,024 | 21.1% | 6,839,895 | 95.0% | 2,262,000 | 38,911,812 | 1,728,426 | 76.4% | 1,728,426 | 76.4% | 1,727,113 | 76% | 38,911,812 | 1,727,113 | 76% | 1,266,720 | 56% | 616 | 1,455,961 | 84% | 1,200,648 | 69.5% | 165,527 | 10% |
| 4100RNA | 30,251,045 | 29,977,518 | 99.1% | 7,935,934 | 26.5% | 7,538,597 | 95.0% | 2,262,000 | 37,603,590 | 1,697,867 | 75.1% | 1,697,867 | 75.1% | 1,696,356 | 75% | 37,603,590 | 1,696,356 | 75% | 990,756 | 44% | 712 | 1,447,957 | 85% | 1,188,812 | 70.1% | 157,395 | 9% |
| 4104RNA | 37,909,124 | 37,687,554 | 99.4% | 5,959,614 | 15.8% | 5,748,830 | 96.5% | 2,262,000 | 38,193,976 | 1,688,271 | 74.6% | 1,688,271 | 74.6% | 1,686,922 | 75% | 38,193,976 | 1,686,922 | 75% | 1,198,860 | 53% | 613 | 1,452,144 | 86% | 1,205,223 | 71.4% | 168,636 | 10% |
| 4108RNA | 33,053,885 | 32,825,688 | 99.3% | 5,432,592 | 16.5% | 5,153,943 | 94.9% | 2,262,000 | 38,541,972 | 1,629,749 | 72.0% | 1,629,749 | 72.0% | 1,628,509 | 72% | 38,541,972 | 1,628,509 | 72% | 1,323,270 | 59% | 706 | 1,454,693 | 89% | 1,179,952 | 72.5% | 166,340 | 10% |
| 4116RNA | 34,896,534 | 34,598,333 | 99.1% | 4,858,396 | 14.0% | 4,491,995 | 92.5% | 2,262,000 | 36,342,480 | 1,643,294 | 72.6% | 1,643,294 | 72.6% | 1,641,794 | 73% | 36,342,480 | 1,641,794 | 73% | 1,253,148 | 55% | 878 | 1,431,630 | 87% | 1,141,458 | 69.5% | 218,860 | 13% |
| 4120RNA | 29,810,797 | 29,606,092 | 99.3% | 5,102,932 | 17.2% | 4,656,255 | 91.2% | 2,262,000 | 33,957,387 | 1,660,701 | 73.4% | 1,660,701 | 73.4% | 1,659,224 | 73% | 33,957,387 | 1,659,224 | 73% | 911,586 | 40% | 827 | 1,431,107 | 86% | 976,580 | 58.9% | 223,538 | 13% |
| 4124RNA | 26,801,585 | 26,624,230 | 99.3% | 3,090,880 | 11.6% | 2,934,018 | 94.9% | 2,262,000 | 34,670,909 | 1,633,386 | 72.2% | 1,633,386 | 72.2% | 1,631,641 | 72% | 34,670,909 | 1,631,641 | 72% | 1,262,196 | 56% | 798 | 1,434,110 | 88% | 998,391 | 61.2% | 190,377 | 12% |
| 4128RNA | 30,427,021 | 30,198,136 | 99.2% | 4,071,868 | 13.5% | 3,828,591 | 94.0% | 2,262,000 | 32,419,126 | 1,627,283 | 71.9% | 1,627,283 | 71.9% | 1,626,040 | 72% | 32,419,126 | 1,626,040 | 72% | 1,045,044 | 46% | 732 | 1,418,905 | 87% | 1,049,321 | 64.5% | 251,144 | 15% |
| 4132RNA | 31,239,710 | 31,030,321 | 99.3% | 4,225,484 | 13.6% | 4,062,390 | 96.1% | 2,262,000 | 32,375,695 | 1,596,689 | 70.6% | 1,596,689 | 70.6% | 1,594,541 | 70% | 32,375,695 | 1,594,541 | 70% | 1,178,502 | 52% | 867 | 1,423,167 | 89% | 978,520 | 61.4% | 212,840 | 13% |
|  |  |  |  |  |  |  |  |  |  |  |  |  |  |  |  |  |  |  |  |  |  |  |  |  |  |  |  |
| min | 26,400,683 | 26,191,989 | 99.0% | 3,090,880 | 11.6% | 2,934,018 | 77.0% | 2,262,000 | 30,684,240 | 1,539,579 | 68.1% | 1,539,579 | 68.1% | 1,537,919 | 68.0% | 30,684,240 | 1,537,919 | 68.0% | 911,586 | 40.3% | 567 | 1,413,144 | 83.9% | 956,765 | 58.9% | 157,395 | 9.2% |
| max | 38,443,941 | 38,122,679 | 99.4% | 9,625,062 | 29.0% | 9,108,755 | 97.9% | 2,262,000 | 40,342,742 | 1,728,426 | 76.4% | 1,728,426 | 76.4% | 1,727,113 | 76.4% | 40,342,742 | 1,727,113 | 76.4% | 1,323,270 | 58.5% | 956 | 1,461,559 | 92.8% | 1,245,282 | 74.0% | 251,144 | 15.4% |
| average | 32,716,305 | 32,478,023 | 99.3% | 5,221,691 | 16.1% | 4,949,635 | 94.9% | 2,262,000 | 35,345,761 | 1,637,811 | 72.4% | 1,637,811 | 72.4% | 1,635,969 | 72.3% | 35,345,761 | 1,635,969 | 72.3% | 1,162,216 | 51.4% | 753 | 1,435,499 | 87.8% | 1,090,878 | 66.6% | 193,700 | 11.9% |
| stdev | 3,357,546 | 3,337,218 | 0.1% | 1,318,165 | 3.8% | 1,251,138 | 3.8% | - | 2,829,476 | 52,068 | 2.3% | 52,068 | 2.3% | 52,128 | 2.3% | 2,829,476 | 52,128 | 2.3% | 114,070 | 5.0% | 103 | 13,854 | 2.2% | 77,833 | 3.7% | 25,690 | 1.8% |
