# Supplementary figures and images for: Metagenomic and metatranscriptomic analysis of saliva reveals disease-associated microbiota in patients with periodontitis and dental caries
Source: NPJ Biofilms Microbiomes. 2017 Oct 2;3:23. doi: 10.1038/s41522-017-0031-4 (PMC5624903; doi:10.1038/s41522-017-0031-4)

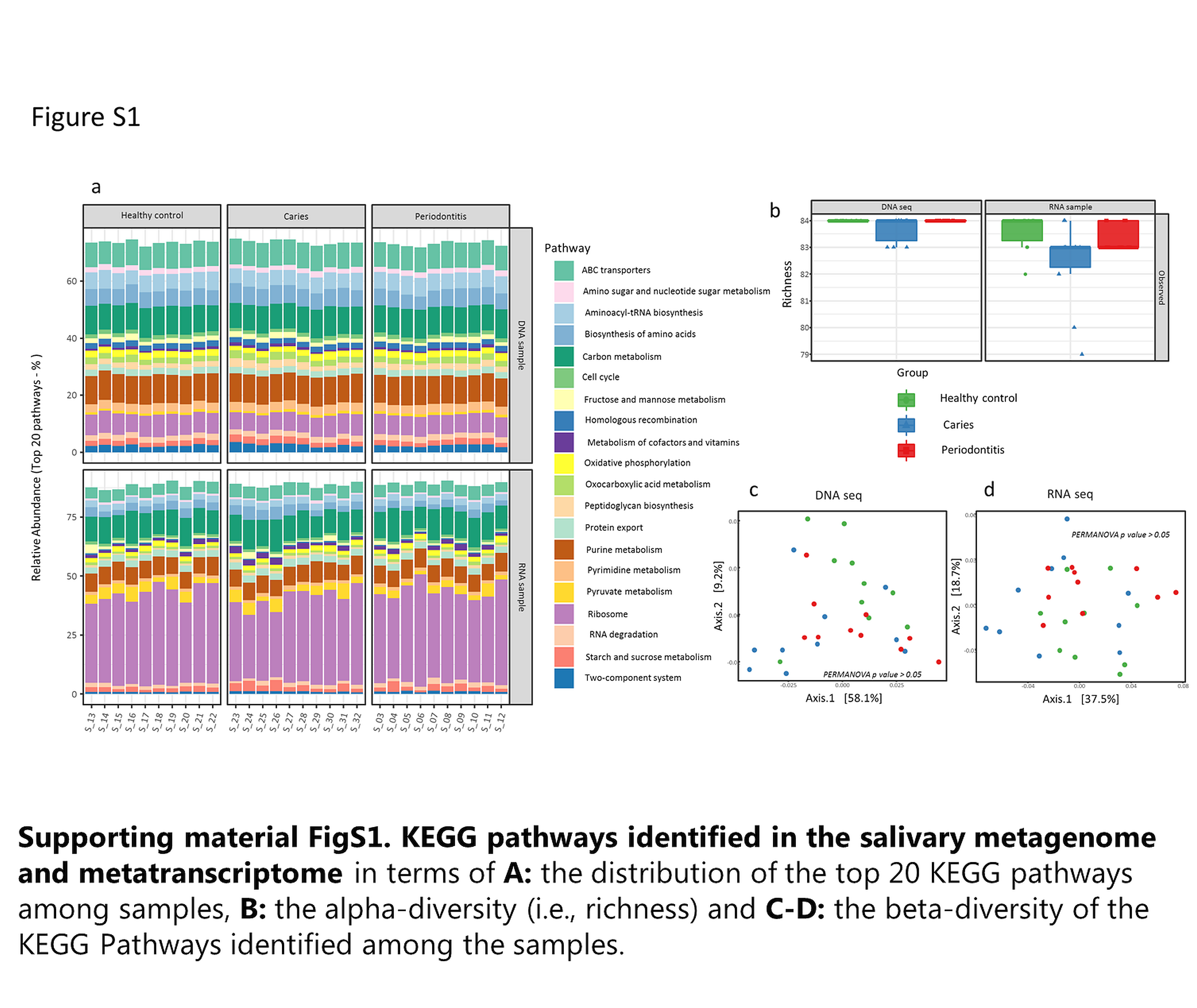

Supplement: Supplementary file 2 — Figure S1 [file 41522_2017_31_MOESM2_ESM.png]

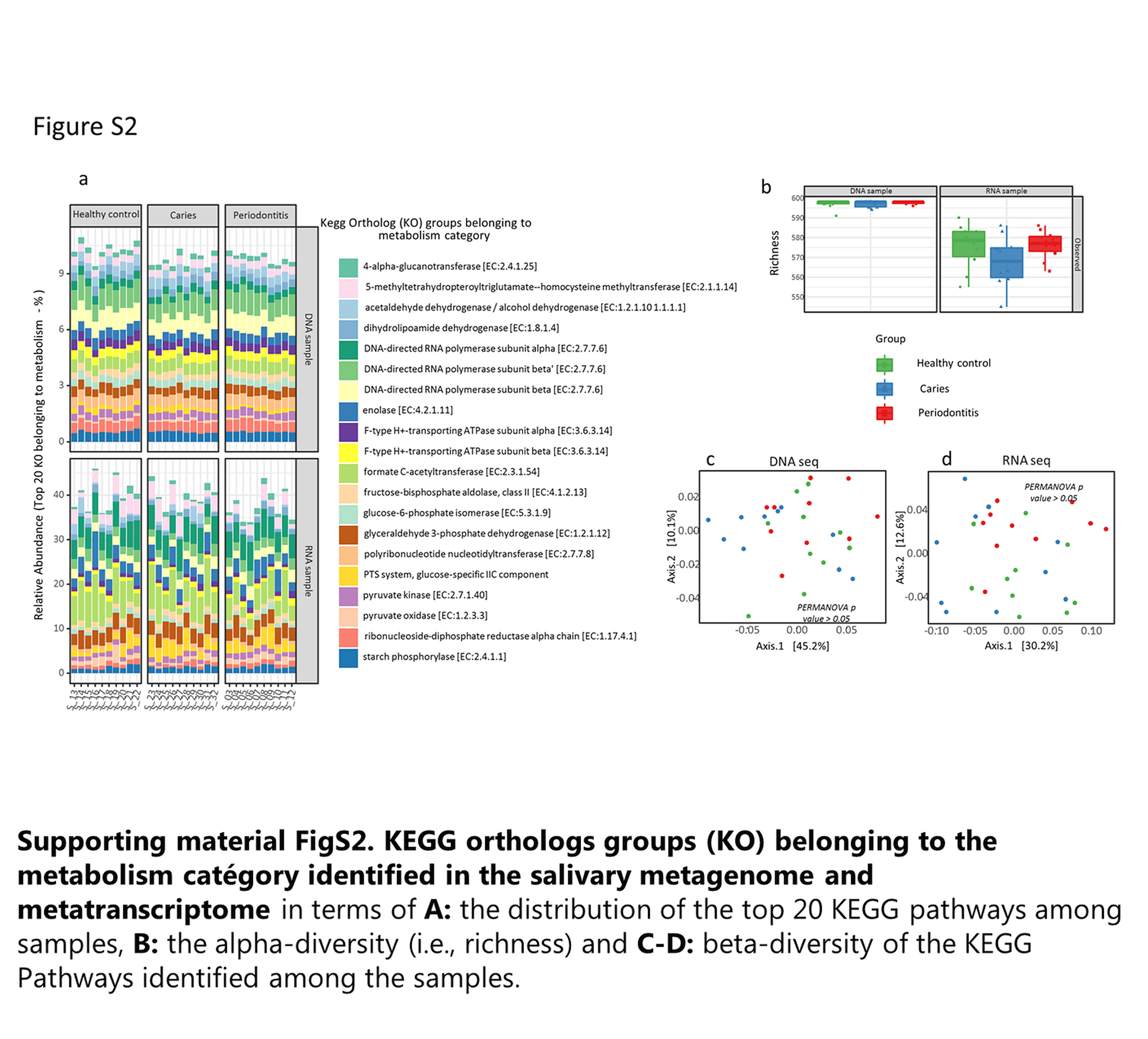

Supplement: Supplementary file 3 — Figure S2 [file 41522_2017_31_MOESM3_ESM.png]
